# Symptoms of Depression and Anxiety During the Early Phase of the COVID-19 Pandemic in Sweden

**DOI:** 10.3389/fpubh.2021.562437

**Published:** 2021-06-02

**Authors:** Elisabet Rondung, Anna Leiler, Jennifer Meurling, Anna Bjärtå

**Affiliations:** Department of Psychology and Social Work, Mid Sweden University, Östersund, Sweden

**Keywords:** COVID-19, pandemic consequences, mental health, depression, anxiety, risk factors

## Abstract

In this cross-sectional study we aimed to assess symptoms of depression and anxiety at an early stage of the COVID-19 pandemic, and to explore factors predictive of these mental health outcomes. A sample of 1,503 participants, recruited from the general Swedish population, completed an online survey distributed through social media. In this sample, 22.2% reported clinically significant levels of depressive symptoms (PHQ-9 ≥ 10) and 10.9% indicated possible major depression using the PHQ-9 algorithm. Moreover, 28.3% reported clinically significant levels of anxiety (GAD-7 ≥ 8) and 9.7% severe anxiety and possible GAD (GAD-7 ≥ 15). Multiple linear regression analyses identified some common predictors for both outcomes. Age, having a stable income, and sufficient social stimulation, sleep, and recovery showed negative associations, whereas worry about the economy and overall burden showed positive associations. These results suggest an impact on mental health already at an early stage of the COVID-19 pandemic.

## Introduction

On December 31, 2019, the World Health Organization (WHO) was informed about pneumonia cases of unknown cause, occurring in the city of Wuhan, China. On January 7, 2020, the cause of the pneumonia was identified as a new type of corona virus, the SARS-CoV-2 (severe acute respiratory syndrome coronavirus 2), and as of January 30, the WHO stated that the virus constituted a public health emergency of international concerns. On March 11, the WHO declared the new coronavirus disease, COVID-19, a pandemic. By then, it had spread to 114 countries, there were more than 120,000 confirmed cases in the world, and about 4,000 people were reported to have died from the disease (1). In Sweden, the first case of COVID-19 was detected in the end of January, and on March 10, the public health agency stated that there were signs of community transmission in Sweden. When this manuscript was first finalized, May 7, 2020, Sweden has almost 25, 000 confirmed cases (applying restricted testing) and over 3 000 fatalities due to COVID-19. This rapid spread of the SARS-CoV-2 and its effects on societies throughout the world, was unprecedented. Although mental health consequences were expected, the timing, extent, and predictors of any such consequences were unknown at the time.

Early on, preliminary results from China, the first country affected by COVID-19, pointed to an increased mental health burden associated with the outbreak of the virus (2–6). However, one study also pointed to positive effects, showing that even though participants experienced a mild increase in stress, they also relaxed and exercised more than usually, received increased social support, and experienced increased feelings of sharing with family members (7).

Since research about the pandemic situation was sparse when this study was conducted, findings from previous epidemics were used to guide ideas about the current situation. For example, when studying the impact of the Ebola outbreak, Jalloh et al. (8) found a high prevalence of any anxiety-depression symptoms and symptoms of posttraumatic stress in the general population of Sierra Leone. Factors associated with higher levels of symptoms included knowing someone quarantined for Ebola and perceiving Ebola as a threat. In a study from the less affected US, Thompson et al. (9) found Ebola-related worry to be positively associated with for example prior mental health diagnoses and high Ebola-related media exposure. However, not all studies pointed to increased levels of mental health symptoms. Studies assessing the psychological impact of the 2002–2004 outbreak of SARS (SARS-CoV-1;10), and H1N1 [or the swine flu; (10)] did not find increased levels of distress, although Ko et al. (11) showed that groups directly affected had more symptoms than non-affected groups.

A review, rapidly conducted in order to better understand effects of being quarantined (published online in late February 2020), showed that most studies on the effects of having been in quarantine reported negative psychological effects, such as trauma and stress-related disorders, anxiety, low mood, irritability, and anger (12). Factors associated with adverse psychological outcomes included both peri-quarantine factors, such as duration of quarantine, fear of infection, frustration and boredom, inadequate supplies and information, and post-quarantine factors, such as stigma and financial loss.

At the time the study was performed, it could only be assume that the new pandemic would lead to financial consequences for many individuals. In Sweden, we started to see increasing rates of unemployment, and many people had been temporarily laid off. Looking back to the financial crisis that started in 2007, research reports suggested that mental health was generally not affected in most European countries (13), but that there was a substantial increase of mental health problems in more affected countries, such as Spain (14). Sweden was more affected by the economic crisis in the nineties, during which a national decrease in psychological well-being also could be seen (15). Thus, any financial consequences of the COVID-19 pandemic were expected to mediate negative effects on mental health.

When this study was performed, the WHO stated that “the overarching goal for all countries is to control the pandemic by slowing down the transmission and reducing mortality associated with COVID-19” [(16); p. 5]. Even though mental health issues, for good reasons, were less prioritized at that time, it is important to understand how psychological well-being is affected by a pandemic situation. With this cross-sectional study, we aimed to assess symptoms of depression and anxiety in the Swedish general population at an early stage of the COVID-19 pandemic. We furthermore aimed to explore factors predictive of these mental health outcomes.

## Materials and Methods

### Design and Setting

This cross-sectional study was conducted at an early stage of the COVID-19 pandemic in Sweden. During a period of 11 days, from March 26 to April 5, 2020, we collected anonymous data online, using the online survey software Qualtrics (Qualtrics; Provo, UT). During this time, the number of confirmed cases in the world went from 531,865 to 1,201,483 and in Sweden from 2,840 to 6,443 (17). At this time, Sweden had no formal movement restrictions, but the public was advised to practice social distancing. Universities and high schools/colleges had closed and applied online teaching, but younger children went to school as usual. On March 13, larger gatherings were restricted to 500 people, and by March 27 this number dropped to a maximum of 50 individuals. People aged 70 or above were recommended to avoid all social contacts. The public health agency also recommended people to avoid traveling within Sweden.

### Sample Size Calculation

Sample size calculations were based on prepandemic data, suggesting a 10.8% prevalence of clinically significant symptoms of depression and a 14.7% prevalence of clinically significant symptoms of anxiety in the general Swedish population (18). With a 2% precision and a 95% confidence interval, a power calculation using http://sampsize.sourceforge.net/ suggested a sample size of 926–1,205. In order to allow the prevalence to raise to ~20%, the sample size was preferred to approach 1,500 participants.

### Procedure

A convenience sampling procedure was used. The study was presented in social media (Facebook, Instagram, and LinkedIn), with a direct link to the survey. We also used an open press release and local radio to spread information about the study. Following the link to the survey, visitors could first read detailed information about the study and their rights as participants. Before entering the questionnaire, participants had to verify being at least 18 years of age (which was our only criteria for eligibility) and give a digital consent to participate in the study. Hence, participants were self-recruited.

The questionnaire was presented in two sections, of which the first covered questions relating to demographics, life style, and COVID-19, and the second focused on issues related to quality of life and mental health. For ethical reasons, any question could be left unanswered.

### Participants

In all, 1,695 eligible individuals gave their consent to participate in the study. Of these, 1,504 (89%) completed the full questionnaire. One participant had taken the questionnaire without giving a single response and was therefore excluded, rendering a final sample of 1,503 participants.

### Variables

#### Outcomes

Two mental health outcomes, symptoms of depression and symptoms of anxiety, were analyzed.

##### Depression

To measure symptoms of depression we used the Patient Health Questionnaire-9, PHQ-9 (19), a self-report instrument used to detect, diagnose, monitor or measure severity of depression. The scale consists of nine items with four labeled response alternatives scored from 0 to 3. The sum of score is used, yielding a maximum score of 27 where higher scores indicate more depressive symptoms. Diagnostic validity has been established, with high sensitivity and specificity in identifying major depressive disorder using a cut-off score of 10 (19, 20). The PHQ-9 is widely used in both clinical and research settings (21), and has previously been used to assess prevalence of depression in the Swedish general population (18). In this study we used the cut point of 10 to indicate clinically significant depressive symptoms, alongside a diagnostic algorithm previously used by Johansson et al. (18), to indicate probable cases of major depressive disorder. In the present sample, Cronbach's α showed a good internal consistency (α = 0.88, *N* = 1,502).

##### Anxiety

We used the General Anxiety Disorder 7, GAD-7 (21) to measure symptoms of anxiety. The GAD-7 was developed as a brief assessment tool for generalized anxiety disorder (GAD) covering several aspects of anxiety and worry. It has the same response system as PHQ-9, and the scale ranges from 0 to 21. Cut-offs for mild (5), moderate (11) and severe (15) anxiety symptoms have been identified, and for diagnosing general anxiety disorder a cut-off of 10 is recommended (22). GAD-7 is well-validated and established in both research and clinical settings (21). Although developed for the disorder GAD, the scale is frequently used to screen for symptoms of anxiety, and anxiety disorders in general. For this purpose a cut-off of 8 has been recommended (23). This cut-off score has previously been used to assess prevalence of clinically significant anxiety in the Swedish general population (18). In the current study, we used a cut point of 8 to indicate clinically significant anxiety symptoms, and the ≥15 cut point to indicate severe anxiety and possible GAD (22). Cronbach's α showed excellent internal consistency (α = 0.91, *N* = 1,502).

#### Predictors

In addition to background characteristics of the participants, we also included context dependent predictors relating to the pandemic situation. Details about the predictors, including response alternatives and variable coding, can be found in Table 1.

**Table 1:** Detailed information about the predictor variables.

**Predictors**	**Item description**
**Background characteristics**	
Age group	Age cohorts: 18–29 [1], 30–39 [2], 40–49 [3], 50–59 [4], 60–69 [5], ≥ 70 [6].
Gender	Male [0], Female [1], Other.
Education	Primary school [1], Gymnasium [2], University 1–3 years [3], University more than 3 years [4].
Occupational status and employment in February 2020	Employed in public sector, Employed in private sector, Self-employed, Student, Retired, On sick-leave, On parental leave, Unemployed, Other; several response alternatives were possible. In the regression analysis all types of employment and being retired were recoded into Stable income [No = 0, Yes = 1].
Number of people in the household	[1–6 or more].
Children <18 years in the household	[No = 0, Yes = 1].
Household responsibility	How would you describe your responsibility for the household you live in? Not responsible [0], Shared responsibility [1], Full responsibility [2].
**Context dependent variables**	
*Risk group*	
Belonging to risk group	Do you have any underlying medical condition that would affect your risk if infected by the Corona virus? (Yes, No, I don't know). This item was coded in combination with the age item into risk group belongingness as follows: No [0], Underlying medical condition, Aged 70 or above [Either or both of these were coded as 1].
Risk group in household	Does anyone else in your household belong to risk group? (No, Yes – due to age, Yes – due to medical condition, I don't know. Several answeres could be given). The item was coded as follows: No [0], Underlying medical condition, Aged 70 or above [Either or both of these were coded as 1].
*Pandemic consequences*	
Avoidance of social contacts	Has the risk of being infected or transmitting the virus to someone else made you avoid other people? Yes, completely [3], Yes, to a large extent [2], Yes, to some extent [1], No, not at all [0].
Duration of social avoidance	If 1–3 on the previous item: For how long have you been avoiding others in this was? A couple of days [1], About 1 week [2], 2 weeks [3], 3 weeks [4], 1 month [5], 1$\frac{1}{2}$ month [6], 2 months or more [7]. If answering Not at all on the previous item, this item was coded as [0].
Negative economic consequences	To what degree have your household suffered from negative economic consequences due to the pandemic? Not at all [0], To a small [1], moderate [2], high [3], and very high degree [4], I don't know.
Worry about economy	To what degree has the pandemic situation made you worry about economic consequences for yourself or someone else in your household? Not at all [0], To a small [1], moderate [2], high [3], and very high degree [4].
Worry about disease	Four items asking for worry about (1) catching the virus, (2) transmitting it to someone close, (3) transmitting it to someone in the social network, and (4) worry about contributing to a general spread. All items had the same response alternatives: Not at all [0], To a small [1], moderate [2], high [3], and very high degree [4]. For the correlation and regression analyses, the mean of the four items was converted to one variable, Worry about the disease [scale item, range 0–4], with an acceptable Chronbach's alpha (α =0.78, *N* = 1,503, M = 1.96, SD = 0.83).
Overall load/burden	How would you describe your daily life burden today, compared with how it was before the pandemic? Considerably lower [1], Somewhat lower [2], No change [3], Somewhat higher [4], and Considerably higher [5] compared to before the pandemic.
*Life style behaviors*	
Current level of social stimulation, Intellectual stimulation, Physical activity, Sleep, and Recovery	To what extent do you in your current daily life experience sufficient (1) social stimulation, (2) intellectual stimulation, (3) physical activity, (4) sleep, and (5) recovery? Not at all [0], To a small [1], moderate [2], high [3], and very high degree [4]. This item was included in the correlation and regression analyses.
Changes in social stimulation, Intellectual stimulation, Physical activity, Sleep, and Recovery	How has your way of living changed since the onset of the pandemic, with regard (1) social stimulation, (2) intellectual stimulation, (3) physical activity, (4) sleep, and (5) recovery? (Considerably less, Somewhat less, No change, Somewhat more, and Considerably more compared to before the pandemic). This item was not included in the correlation and regression analyses.
*Information and trust*	
Time spent on information	How much time do you spend on taking part of information about the virus and its consequences? (None [0], <1 h [1], 1–2 h [2], 2–3 h [3], 3–4 h [4], 4–5 h [5], 5–10 h [6] more than 10 h daily [7]).
Trust in authorities	To what extent do you experience trust in the authorities' capacity to handle the situation? Not at all [0], To a small [1], moderate [2], high [3], and very high degree [4].

*Dummy variables encoded for regression analysis are shown in square brackets*.

The context dependent variables were categorized in four groups. The first group included questions about if the participant or any household member belonged to a known *risk group* for COVID-19. The second group of context dependent variables focused on *pandemic consequences*. In this group we included questions about avoidance of social contacts, negative economic consequences or worry about such consequences, worry about the disease, and changes in the daily life burden. In the third group we assembled items asking about *life style behaviors*. Here we used two sets of life style questions, each with five items. In the first set of questions, participants were asked if they, under the current circumstances, experienced sufficient social stimulation, intellectual stimulation, physical activity, sleep, and recovery. These items were included in the correlation and regression analyses. In the second set of items, the current levels were to be compared with the situation before the pandemic onset. Finally, in the fourth group of context dependent predictors, *information and trust*, we asked participants to rate the time spent on information about the new virus and its consequences and their trust in authorities' capacity to handle the situation. Although not specified in the questionnaire, relevant Swedish authorities were for example the Swedish Government and the Public Health Agency of Sweden.

### Data Analysis

Data was analyzed using IBM SPSS Statistics 25. First we deleted participants who had not completed the full survey. The remaining 1,503 participants were included in the analysis. With regard to the symptom scales, we used individual mean imputation to correct for item non-responses not exceeding 20% of a particular scale (24). Participants with a higher rate of missing scale items were excluded scale wise, leading to 1,502 valid cases for both PHQ-9 and GAD-7.

In order to describe the study sample, we used frequencies and proportions with 95% confidence intervals (CI) for nominal and ordinal variables. For the outcome variables, we used descriptive statistics in terms of means with 95% CIs and standard deviations, displayed for the complete sample, for the different age groups, and for men and women in each age group separately. To enable comparisons with previous studies conducted in Sweden, we also calculated the prevalence of clinical levels of depressive and anxiety symptoms, using previously known cut off points and diagnostic algorithms (PHQ-9 ≥ 10, PHQ-9 algorithm, GAD-7 ≥ 8, and GAD-7 ≥ 15).

We thereafter explored the dataset in order to investigate patterns of correlations with the outcome variables. Intercorrelations between predictor variables were also inspected to circumvent multicollinearity. In order to target the most important predictive factors for symptoms of depression and anxiety we performed one multiple linear regression for each outcome, using forced entry of all predictors that correlated significantly with each particular outcome. Having a large sample with several predictors, a significant level of 1% or smaller was adopted.

In order to shed further light on predictors, the regression analyses were followed by descriptive and/or exploratory analyses of key predictor variables. Since these analyses were guided by previous findings, statistical methods are briefly motivated in the Results section, together with a presentation of the most important results.

## Results

### Sample Characteristics

In this sample, we had participants in all age groups, from 18–29 years of age, to 80 years or older. Since participants in the older age groups were few, all participants over 70 years of age were collapsed into one age group. The sample was characterized by a clear majority of female participants (82%) and a generally high level of education (see Table 2 for demographic information).

**Table 2:** Characteristics of survey participants (*N* = 1,503).

**Characteristic**	***n***	***%***
**Age**		
18–29	183	12.2
30–39	368	24.5
40–49	352	23.4
50–59	313	20.8
60–69	189	12.6
≥ 70	98	6.5
**Gender**		
Female	1,232	82.0
Male	261	17.4
Other	10	0.7
**Level of education**		
Primary school	24	1.6
High school	263	17.5
University 1–3 years	322	21.4
University >3 years	893	59.4
Response missing	1	0.1
**Occupational status** *(Several responses possible)*		
Employed in public sector	720	47.9
Employed in private sector	324	21.6
Self-employed	113	7.5
Student	187	12.4
Retired	185	12.3
On sick-leave	59	3.9
On parental leave	29	1.9
Unemployed	62	4.1
Other	39	2.6
**Number of people in household**		
1	385	25.6
2	439	29.2
3	251	16.7
4	287	19.1
5	91	6.1
≥6	32	2.1
Response missing	18	1.2
**Role in household**		
Shared responsibility for household	977	65.0
Sole responsibility for household	462	30.7
Someone else is responsible	59	3.9
Response missing	5	0.3
**Children in household** *(Several responses possible)*		
No children	912	60.7
Children <1 year of age	38	2.5
Children aged 1–5	182	12.1
Children aged 6–11	270	18.0
Children aged 12–17	300	20.0
Response missing	4	0.3
**Participants belonging to a risk group**		
70 years of age or more^a^	98	6.5
Medical risk group^b^	313	20.8
Unsure of medical risk	80	5.3
Double risk groups (≥70 years and medical risk group)	45	3.0
No risk group	1,061	70.6
**Household member belonging to a risk group** *(Several responses possible)*		
70 years of age or more^a^	122	8.1
Medical risk group^b^	264	17.6
Unsure of medical risk	51	3.4
Double risk groups (≥70 years and medical risk group)	56	3.7
No risk group	1,131	75.2
Response missing	1	0.1

a *In Sweden, people over 70 years of age were defined as belonging to a risk group at this time*.

b *Do you have any underlying disease (such as high blood pressure, cardiovascular disease, lung disease, cancer or diabetes) that affects your risk if infected by the new coronavirus?*

### Symptoms of Depression and Anxiety

Symptoms of depression (*M* = 6.24, 95% CI 5.96–6.53) and anxiety (*M* = 5.73, 95% CI 5.46–6.00) were highly correlated, *r*(1,501) = 0.77 (95% CI 0.73–0.80). Descriptive statistics of the two outcomes are presented in Table 3. Data from this sample showed a trend of decreasing symptom load with increasing age.

**Table 3:** Descriptive statistics of mental health variables displayed per age group (*N* = 1,503).

	**Depression PHQ-9**			**Anxiety GAD-7**		
**Age group (valid responses)**	***M***	***(SD)***	***95% CI***	***M***	***(SD)***	***95% CI***
**18–29 years (*****n*** **= 183)**	**8.55**	**(5.95)**	**7.68–9.41**	**8.02**	**(5.59)**	**7.20–8.83**
Women (*n* = 138)	8.38	(6.01)	7.37–9.40	8.07	(5.63)	7.13–9.02
Men (*n* = 38)	8.61	(5.90)	6.67–10.54	7.24	(5.28)	5.50–8.97
**30–39 years (*****n*** **= 367)**	**6.88**	**(5.56)**	**6.31–7.45**	**6.85**	**(5.75)**	**6.26–7.44**
Women (*n* = 304)	6.95	(5.52)	6.32–7.57	7.07	(5.70)	6.43–7.72
Men (*n* = 60)	6.30	(5.23)	4.94–7.65	5.63	(5.73)	4.15–7.11
**40–49 years (n = 352)**	**6.46**	**(5.62)**	**5.87–7.05**	**5.87**	**(5.21)**	**5.32–6.41**
Women (*n* = 292)	6.33	(5.47)	5.70–6.96	5.78	(5.23)	5.18–6.38
Men (*n* = 60)	7.10	(6.29)	5.47–8.73	6.30	(5.16)	4.97–7.63
**50–59 years (n = 312)**	**5.57**	**(5.52)**	**4.96–6.19**	**4.63**	**(4.74)**	**4.10–5.16**
Women (*n* = 263)	5.55	(5.54)	4.88–6.22	4.79	(4.89)	4.20–5.39
Men (*n* = 49)	5.69	(5.47)	4.12–7.26	3.76	(3.77)	2.67–4.84
**60–69 years (n = 188)**	**4.66**	**(5.15)**	**3.92–5.40**	**4.10**	**(4.71)**	**3.43–4.78**
Women (*n* = 160)	4.73	(4.97)	3.96–5.51	4.26	(4.78)	3.51–5.01
Men (*n* = 28)	4.21	(6.13)	1.84–6.59	3.21	(4.25)	1.56–4.86
**70 years or more (*****n*** **= 98)**	**3.93**	**(3.83)**	**3.16–4.70**	**3.32**	**(3.56)**	**2.61–4.04**
Women (*n* = 73)	4.55	(4.02)	3.61–5.49	4.02	(3.71)	3.16–4.89
Men (*n*= 25)	2.12	(2.51)	1.09–3.15	1.28	(2.01)	0.45–2.11
***Total sample (N = 1502)***	***6.24***	***(5.60)***	***5.96–6.53***	***5.73***	***(5.33)***	***5.46–6.00***
***Women (n = 1232)***	***6.23***	***(5.52)***	***5.92–6.54***	***5.84***	***(5.34)***	***5.54–6.14***
***Men (n= 261)***	***6.06***	***(5.78)***	***5.36–6.77***	***4.99***	***(5.06)***	***4.37–5.61***

*PHQ-9: 9-item Patient Health Questionnaire Depression Scale; GAD-7: 7-item Generalized Anxiety Disorder Scale; 95% CI: 95% Confidence Intervals*.

The overall prevalence of clinically significant depression (PHQ-9 ≥ 10) was 22.2% (334/1,502, 95% CI 20.2–24.4), and the PHQ-9 algorithm indicated major depression in 10.9% (164/1,502, 95% CI 9.4–12.6). The prevalence of clinically significant levels of anxiety (GAD-7 ≥ 8) was 28.3% (425/1,502, 95% CI 26.0–30.6), whereas the prevalence of severe anxiety and possible GAD (GAD-7 ≥ 15) was 9.7% (145/1,502, 95% CI 8.2–11.3). The pattern of decreasing symptoms with increased age was once again suggested, with all prevalence ratings decreasing from younger to older age groups (see Table 4).

**Table 4:** Prevalence (%) of depression and anxiety in different age groups (*n* = 1,503).

	**Clinically significant depressive symptoms**		**Possible major depression**		**Clinically significant Anxiety symptoms**		**Severe anxiety, possible GAD**	
**Age group (valid responses)**	**(PHQ-9 ≥ 10)**		**(PHQ-9 algorithm)**		**(GAD-7 ≥ 8)**		**(GAD-7 ≥ 15)**	
**18–29 years (*****n*** **= 183)**	**39.3**	**(32.2–46.8)**	**18.0**	**(12.8–24.4)**	**45.4**	**(38.0–52.9)**	**15.8**	**(10.9–22.0)**
Women (*n* = 138)	37.7	(29.6–46.3)	18.8	(12.7–26.4)	45.7	(37.2–54.3)	16.7	(10.9–24.0)
Men (*n* = 38)	42.1	(26.3–59.2)	13.2	(4.4–28.1)	39.5	(24.0–56.6)	10.5	(2.9–24.8)
**30–39 years (*****n*** **= 368)**	**25.8**	**(21.4–30.6)**	**11.7**	**(8.6–15.4)**	**34.5**	**(29.7–39.6)**	**13.9**	**(10.5–17.8)**
Women (*n* = 305)	25.6	(20.8–30.9)	11.5	(8.1–15.6)	36.4	(31.0–42.1)	14.4	(10.7–18.9)
Men (*n* = 60)	26.7	(16.1–39.7)	11.7	(4.8–22.6)	25.0	(14.7–37.9)	10.0	(3.8–20.5)
**40–49 years (*****n*** **= 352)**	**22.4**	**(18.2–27.2)**	**12.8**	**(9.5–16.7)**	**27.8**	**(23.2–32.8)**	**10.2**	**(7.3–13.9)**
Women (*n* = 292)	19.5	(15.1–24.5)	11.0	(7.6–15.1)	26.0	(21.1–31.5)	10.3	(7.0–14.3)
Men (*n* = 60)	36.7	(24.6–50.1)	21.7	(12.1–34.2)	36.7	(24.6–50.1)	10.0	(3.3–20.5)
**50–59 years (*****n*** **= 312)**	**16.9**	**(12.9–21.6)**	**8.9**	**(6.0–12.7)**	**22.4**	**(17.9–27.5)**	**6.1**	**(3.7–9.3)**
Women (*n* = 263)	17.5	(13.1–22.6)	8.4	(5.3–12.4)	23.6	(18.6–29.2)	6.5	(3.8–10.1)
Men (*n* = 50)	14.0	(5.8–26.7)	12.0	(4.5–24.3)	16.3	(7.3–29.7)	4.1	(0.5–14.0)
**60–69 years (*****n*** **= 189)**	**13.8**	**(9.2–19.6)**	**6.9**	**(3.7–11.5)**	**17.5**	**(12.3–23.6)**	**5.3**	**(2.6–9.5)**
Women (*n* = 160)	14.4	(9.3–20.8)	6.2	(3.0–11.1)	18.6	(12.9–25.5)	5.6	(2.6–10.3)
Men (*n* = 28)	10.7	(2.3–28.2)	10.7	(2.3–28.2)	10.7	(2.3–28.2)	3.6	(0.1–18.3)
**70 years or more (*****n*** **= 98)**	**9.2**	**(4.3–16.7)**	**2.0**	**(0.2–7.2)**	**14.3**	**(0.8–22.8)**	**0.0**	**(0.0–3.7)**
Women (*n* = 73)	12.3	(5.8–22.1)	2.7	(0.3–9.5)	17.8	(9.8–28.5)	0.0	(0.0–4.9)
Men (*n* = 25)	0.0	(0.0–13.7)	0.0	(0.0–13.7)	0.4	(0.1–20.4)	0.0	(0.0–13.7)

*95% Confidence Intervals are given within parenthesis. GAD, Generalized Anxiety Disorder; PHQ-9: 9-item Patient Health Questionnaire Depression Scale; GAD-7: 7-item Generalized Anxiety Disorder Scale*.

### Predictors of Mental Health

The outcome variables showed no correlations with the gender item, number of people in the household, living with children under 18 years of age, household responsibility, or any of the risk group variables (see Table 5). Hence, these variables were removed from further analysis.

**Table 5:** Significant Pearson's correlations between outcomes and predictors.

		**Depression PHQ-9**	**Anxiety GAD-7**
**Predictors**	**Valid *N***	***r***	***r***
*Background Characteristics*			
Group	1,502	−0.22^**^	−0.26^**^
Gender	1,492^a^	0.01	0.06
Education	1,501	−0.17^**^	−0.11^**^
Stable income	1,502	−0.22^**^	−0.18^**^
Number of people in the household	1,484	0.01	0.02
Children <18 years	1,498	<0.01	0.06
Household responsibility	1,497	0.05	−0.03
*Context dependent variables*			
*Risk group*			
Belonging to risk group	1,422^b^	0.05	−0.02
Risk group in household	1,454^b^	<0.01	−0.01
*Pandemic consequences*			
Avoidance of social contacts	1,502	0.15^**^	0.20^**^
Duration of social avoidance	1,502^c^	0.07^*^	0.11^**^
Negative economic consequences	1,450-51^b^	0.22^**^	0.21^**^
Worry about economy	1,502-03	0.33^**^	0.35^**^
Worry about disease	1,502	0.24^**^	0.37^**^
Overall load/burden	1,501	0.28^**^	−0.31^**^
*Life style behaviors*			
Social stimulation	1,501	−0.30^**^	−0.27^**^
Intellectual stimulation	1,501	−0.35^**^	−0.29^**^
Physical activity	1,500	−0.34^**^	−0.25^**^
Sleep	1,502	−0.39^**^	−0.34^**^
Recovery	1,500	−0.42^**^	−0.42^**^
*Information and trust*			
Time spent on information	1,501	0.15^**^	0.20^**^
Trust in authorities	1,502	0.24^**^	−0.23^**^

* *p ≤ 0.01;*

** *p ≤ 0.001.*

a *The response Other (n = 10) was removed from the present analysis*.

b *The response I don't know (n = 65) was removed from the present analysis*.

c *People who did not isolate to some degree was coded as 0*.

Residual variance around the regression line for bivariate correlations were visually inspected. No outliers with potential to drive the regression line was identified. Multivariate outliers were visually inspected by plotting individual DfFit values, showing a nicely centered fit of the data. Collinearity statistics showed no risk values of variance inflated factor (VIF), nor any correlations above moderate levels between any two predictors. In Table 6, we present beta weights (*B*), standard errors (*SE*), and adjusted beta (β) for all predictors in the two regression analyses, respectively.

**Table 6:** Linear regression for symptoms of depression (PHQ-9), symptoms of anxiety (GAD-7).

	**Depression PHQ-9**		**Anxiety GAD-7**	
**Predictors**	***B* (*SE*)**	**β**	***B* (*SE*)**	**β**
***Background variables***				
Age Group	−0.45 (0.09)^**^	**−0.11**	−0.65 (0.08)^**^	**−0.18**
Education	−0.46 (0.15)^*^	**−0.07**	−0.20 (0.14)	−0.03
Stable income (No = 0)	−1.78 (0.34)^**^	**−0.11**	−1.28 (0.32)^**^	**−0.09**
***Context dependent variables***				
*Pandemic consequences*				
Avoidance of social contacts	0.38 (0.19)	0.05	0.70 (0.18)^**^	**0.09**
Duration of social avoidance	−0.05 (0.11)	−0.01	−0.03 (0.10)	< −0.01
Negative economic consequences	0.19 (0.13)	0.04	0.14 (0.12)	0.03
Worry about economy	0.72 (0.12)^**^	**0.15**	0.67 (0.11)^**^	**0.15**
Worry about disease	0.33 (0.15)	0.05	1.10 (0.14)^**^	**0.17**
Overall load/burden	0.65 (0.12)^**^	**0.12**	0.70 (0.11)^**^	**0.14**
*Life style behaviors*				
Social stimulation	−0.61 (0.15)^**^	**−0.11**	−0.58 (0.14)^**^	**−0.11**
Intellectual stimulation	−0.56 (0.15)^**^	**−0.09**	−0.38 (0.14)	−0.07
Physical activity	−0.48 (0.13)^**^	**−0.09**	0.10 (0.12)	0.02
Sleep	−0.98 (0.15)^**^	**−0.17**	−0.60 (0.15)^**^	**−0.11**
Recovery	−0.75 (0.16)^**^	**−0.13**	−0.89 (0.15)^**^	**−0.17**
*Information and trust*				
Time spent on information	0.25 (0.10)	0.05	0.41 (0.10)^**^	**0.09**
Trust in authorities	−0.37 (0.13)^*^	**−0.06**	−0.25 (0.12)	−0.04

*Standardized beta values of significant predictors (p ≤ 0.01) in bold.*

* *p ≤ 0.01*,

** *p ≤ 0.001*.

#### Depressive Symptoms

The multiple linear regression for depressive symptoms (PHQ-9 *M* = 6.19, *SD* = 5.58) was conducted on responses from 1,445 participants. The analysis showed a significant ANOVA, *F*_(161,428)_ = 62.51, *p* < 0.001, *R* = 0.64, with an adjusted *R*^2^ showing an explained variance of 41%. The most important backgrounds factors were age (β = −0.11) and whether participants had a stable income or not (β = −0.11), indicating that symptoms of depression were higher among younger participants and among participants without a stable income. The most important contextual variables contributing to the model were worry about the economy (β = 0.15), all five lifestyle behaviors (foremost sleep β = −0.17, recovery β = −0.13, and social stimulation β = −0.11), and overall increase in burden (β = 0.12, see Table 6).

#### Anxiety Symptoms

The model with the anxiety scores (GAD-7 *M* = 5.64, *SD* = 5.28, *N* = 1,445) was significant as well, *F*_(161,428)_ = 64.84, *p* < 0.001, *R* = 0.65, adjusted *R*^2^ = 0.41. A similar pattern was shown with age group being the most important background variable (β = −0.18). However, worry about the disease, which was not significant for the depression scores, was one of the most important context dependent predictors (β = 0.17), together with recovery (β = 0.17), worry about the economy (β = 0.15), and overall increase in burden (β = 0.14, see Table 6). Moreover, the degree to which people were isolating from others and time spent seeking information about the pandemic also came forth in this model (β = 0.09 for both predictors, respectively).

### Further Exploration of Predictor Variables

#### Worry About the Disease

As could be anticipated, worry about the disease predicted symptoms of anxiety. We further wanted to explore if there were differences in what participants were worrying about. The four independent items of the worry about the disease variable (worry about getting the disease, worry about infecting someone close, someone in the social network, or contributing to a general spread if the disease) were analyzed with a one way dependent ANOVA followed by Bonferroni corrected pairwise comparisons. The results showed significant differences between the items, *F*_(3,4497)_ = 172.76, *p* < 0.001, η^2^ = 0.10, with respondents showing the largest worry about infecting someone close (*M* = 2.32, *SD* = 1.13) compared to both worry about infecting others in the social network (*M* = 1.91, *SD* = 1.01, *p* < 0.001), and spreading the disease in general (*M* = 1.94, *SD* = 1.01, *p* < 0.001). There was no significant difference between the two latter. However, participants worried the least about getting infected by the virus themselves (*M* = 1.69, *SD* = 0.99, all comparisons *p* < 0.001).

#### Burden and Avoidance of Social Contacts

In this sample, 18.6% described their everyday life as considerably (4.7%) or somewhat (14.0%) less burdened than before the pandemic, and 28.3% described it as more or less the same as before. More than half of the participants (53.0%) described an everyday burden that was somewhat (36.7%) or considerably (16.3%) higher than before the virus outbreak.

Only a few people indicated that they did not avoid social contacts to any degree due to the pandemic (4.3%), while 40.5% avoided others to some degree and 47.8% to a large degree. The remaining 7.5% practiced full avoidance of social contacts.

#### Economy

Negative economic consequences was not a significant predictor in the regression analyses. One explanation could be that the majority of the sample (45.7%) was still unaffected, 24.9% was affected to a small degree, 16.7% to a moderate degree, and 6.5% and 2.7% to a high or very high degree, respectively. In order to give an indication of any difference in symptoms over response categories, Kruskal-Wallis test was used. The results showed increasing symptoms over response categories (indicating more negative economic consequences), for both depression, χ^2^(4) = 69.56, *p* < 0.001, and anxiety, χ^2^(4) = 59.16, *p* < 0.001. Mean score differences in depressive symptoms, for not at all affected (*M* = 5.27, *SD* = 5.10) and affected to a very high degree (*M* = 11.90, *SD* = 7.80), as well as in anxiety (*M* = 4.71, *SD* = 4.83 and *M* = 10.32, *SD* = 7.46, respectively) were evident.

The impact of economic factors was even more evident in worry about the economy, which contributed significantly to both outcomes. In the follow up analyses, we explored if worry about the economy was affected by participants' occupational status and type of employment. For this purpose, the worry variable was treated like a scale variable (0–4), and multiple responses in the occupational variable were prioritized in the following order: 1. retired, 2. student, 3. self-employed, 4. employed in private sector, 5. employed in public sector, 6. on sick leave or parental leave without other occupation, 7. other employment, 8. Unemployed. Univariate ANOVA showed a small but significant effect, *F*_(7,1495)_ = 8.21, *p* < 0.001, η^2^ = 0.04. Bonferroni corrected pairwise comparisons indicated that individuals that were retired (*M* = 1.25, *SD* = 1.12) or employed in the public sector (*M* = 1.53, *SD* = 1.11) were less worried than individuals that were self-employed (*M* = 2.09, *SD* = 1.128, *p* < 0.001) or employed in the private sector (*M* = 1.83, *SD* = 1.18, *p* < 0.001 and *p* < 0.01, respectively). Retired participants were also less worried than students (*M* = 1.705, *SD* = 1.14, *p* = 0.005).

#### Changes in Life Style Behaviors

Among the life-style factors, self-reported changes following the pandemic situation was most evident in participants' social lives. Eighty-two percent of the sample reported some to considerable decrease in social stimulation (37.9 and 44.0% respectively), whereas the corresponding number was lower for all other aspects (intellectual stimulation 42.0%, physical activity 49.9 %, sleep 23.6%, and recovery 24.0 %).

#### Information and Trust

Almost all participants in this sample were actively taking part of information about the disease and its consequences. Only 1.1% did not spend any time at all taking part of information of this kind. Most participants spent <3 h a day on information (32.5% spent <1 h, 40.0% 1–2 h, and 16.5% 2–3 h), while 7.9% spent 3–5 h, and 1.9% of the sample spent >5 h daily on pandemic information.

The participants in this sample generally reported high levels of trust in the authorities' capability of handling the situation. As many as 71.1% reported high (44.9%) or very high (26.1%) trust in the authorities. Another 20.8% reported moderate levels of trust, and just above eight percent reported low (6.5%) or no (1.6%) trust in the authorities.

## Discussion

### Depression and Anxiety

#### Prevalence

In our sample, 22.2% reported clinically significant levels of depressive symptoms (PHQ-9 ≥ 10) and 10.9% indicated possible major depression using the PHQ-9 algorithm. Moreover, 28.3% reported clinically significant levels of anxiety (GAD-7 ≥ 8). When this manuscript was first submitted, very few European studies on mental health during the early phase of COVID-19 pandemic were published. Hence, the high level of symptomatology indicated by our data took us by surprise. In comparison with pre-pandemic Swedish prevalence estimates (18), our results show that clinically significant symptoms of both depression and anxiety are approximately twice as common, and the prevalence is also considerably higher than global pre-pandemic prevalence estimates (25). Although available data from China indicated similar mean levels of anxiety and depression (3), cultural differences made comparisons difficult. However, later studies have confirmed the deterioration of mental health during this phase. For example, Pierce et al. (26) have shown that the population prevalence of clinically significant levels of mental distress in the UK rose from 18.9% in 2018–19 to 27.3% in April, 2020. Also, Swedish data from McCracken et al. (27) confirm the high levels of clinically significant symptoms in our data. With these results on hand, the high levels of symptomatology are in line with the latest research.

#### Predictive Factors

A crisis like this might not affect all parts of the population in the same way. This too has become evident in plural studies published during the last months. These studies have typically found female gender and young age to be risk factors for experiencing anxiety and depression in the early phases of the pandemic (26–29). With regard to gender differences, the disproportionately few men in our sample made us choose not to make statistical comparisons between the genders, but an inspection of the descriptive statistics suggest that we might have caught the expected gender pattern in symptoms of anxiety, although not in depression. In line with others (26–29), younger age did however turn out to be a significant predictor of both anxiety and depression. Although we can only speculate in the reasons for this, reduced social interaction and increased worry might play a role. For example, results from a Belgian study (30) have shown that a decrease in going out for drinks or food was associated with increased mental distress among young people during the pandemic. Based on this finding, the authors discuss the importance of peer interaction for the mental health of the young. In another study, exploratory analyses of students' social networks and mental health before and during the pandemic, have shown that students did not only report more stress, depression and anxiety after the onset of the pandemic, but also more social isolation and loneliness, along with increased worry about their family, friends, own health, economy and future career (31). Hence, young people might be extra vulnerable to the mental health consequences of the pandemic for a larger variety of reasons.

The importance of social interaction was verified in other parts of our results. Only 4% of our sample indicated that they had not avoided social contacts to any extent, and more than 80% reported reduced social stimulation. Social stimulation was also one of the most important predictors of both outcomes. These findings match previously published results, for example U.S. findings from a similar period of time showing that personal distancing and orders of staying at home was associated with higher levels of anxiety and depression (32). In the older parts of the population, staying at home and reducing face-to-face interaction with other people (or “cocooning”) has been associated with reporting worse mental health, worse physical health, and reduced quality of life (33). Taken together, avoidance of social interactions and/or reduced social stimulation seem to be important parameters to consider in understanding mental health in pandemic contexts. With sleep, recovery, intellectual stimulation and physical activity also contributing to the regression models, it is clear that life style variables are of importance, also in a crisis like this.

Another evident characteristic of the ongoing crisis is its consequences for the economy. In this early stage of the pandemic, few people had experienced severe negative economical effects. However, people highly affected showed mean symptom ratings above diagnostic cutoffs for both depression and anxiety, and more than twice as high compared to people unaffected. Even though sample sizes were very unequal and the variance was rather high, this gave an indication of an association that later has been confirmed. For example, Witteveen and Velthorst have shown that a sudden loss of income during the pandemic lockdown almost doubled the risk of depressive feelings (34). Our results also show that two of the economic predictors (not having a stable income and worry about the economy) were important predictors for both depressive and anxiety symptoms. In line with previous results associating economic hardship to mental ill-health (14), economy seem to be an important factor for mental health also during this crisis.

Also, worry about the disease itself showed to be of importance, especially in predicting symptoms of anxiety. Interestingly, our participants were more worried about spreading the disease to others, especially close ones, than about being infected themselves.

We finally had a look at the role of information and media consumption. In line with lessons learned from the Ebola outbreak, where extensive Ebola related media exposure was an important predictor of distress (9), we found that spending more time on information predicted higher levels of anxiety. Similar findings have been shown by others (35, 36).

### Strengths and Limitations

In interpreting the findings from this study, several methodological limitations need to be considered. Given the rapidly changing circumstances, we aimed for a brief time window for data collection. We also aimed to launch the survey as soon as possible, in order to capture mental health in the early phases of the pandemic. To ensure a rapid distribution of the survey, we chose to advertise primarily in social media. However, since we expected that this might skew the sample in a younger direction, we also spread information about the study in other channels that were thought to attract an older audience.

Despite these efforts, our sample was not representative of the Swedish population. With an overwhelming majority of female participants (82%) along with underrepresentation of older, and to some extent also younger participants, great caution is needed when interpreting and generalizing the findings. Without dismissing the possibility of female overrepresentation contributing to the high level of symptoms found, we acknowledge that the prevalence rates identified here are still higher than previous findings among women (18), suggesting that the uneven gender distribution alone is unlikely to account for the discrepancy with previous findings.

Self-selection of participants may also have contributed to the overall high levels of anxiety and depression. It is possible that individuals with mental ill-health are more interested in sharing their experiences by participating in a study like this. It is also likely that the older participants in our sample were healthier and more active than could be expected from a random population sample of the same age. Since they found the survey through social media, they might also be more socially active. This might have contributed to the low levels of depression and anxiety in the oldest age group.

The limitations of a cross-sectional design also need to be taken into account, since a design like this does not support inferences regarding causality or the development of symptoms over time.

## Conclusion and Future Suggestions

With these limitations in mind, we still believe that the current study can contribute to the ongoing exploration of mental health consequences of the COVID-19 pandemic. When initiating this study, it was one of few investigating predictors of mental health in the context of the pandemic in Sweden. Since then, many of our findings have been verified. However, in order to fully understand the mental health consequences of the current crisis, and to guide both future research and societal policies, every piece of information can be of value. This paper adds to the literature exploring a wide range of possible predictors of mental health during the pandemic, primarily showing the influence of age, life style behaviors and worry. These findings could form the basis for studies developing and evaluating interventions to improve mental health among vulnerable groups. Our study showed that symptom burden varied with several background factors such as age and income, suggesting that interventions could be tailored to the varying needs in different groups. However, since factors such as sleep and lack of recovery also showed an association with increased levels of symptoms, general health promoting strategies may also be beneficial and evaluated.

Based on our results, we urge policy makers to promote and enable safe social activities. The fact that young age was associated with a heavier symptom burden indicates that this may be of special importance for young individuals. The positive association between economic worries and distress, as well as the buffering effect that having a stable income seemed to have, further point to the importance of economic support to individuals and companies affected by pandemic related economic difficulties.

## Data Availability Statement

The datasets presented in this article are not readily available because preparation of additional articles using these data is currently in progress. Requests to access the datasets should be directed to Elisabet Rondung, elisabet.rondung@miun.se.

## Ethics Statement

Since we did not collect any data that could be used to identify the participants, ethical review and approval was not required for the study on human participants in accordance with the local legislation and institutional requirements. Informed consent for participation was not required to be written for this study in accordance with the national legislation and the institutional requirements. However, a digital informed consent was obtained.

## Author Contributions

ER and AB constructed the online survey, performed data analyses, and interpreted the findings. AL and ER conducted the literature search and reviewed previous research. ER led the manuscript work, in which AL, JM, and AB made substantive contributions in both drafting and by critical revisions. All authors contributed to the study design and development of the study questionnaire. All authors approved the final version of the manuscript for submission.

## Conflict of Interest

The authors declare that the research was conducted in the absence of any commercial or financial relationships that could be construed as a potential conflict of interest.
